# Linking the Tinnitus Questionnaire and the subjective Clinical Global Impression: Which differences are clinically important?

**DOI:** 10.1186/1477-7525-10-79

**Published:** 2012-07-10

**Authors:** Ilya Adamchic, Peter Alexander Tass, Berthold Langguth, Christian Hauptmann, Michael Koller, Martin Schecklmann, Florian Zeman, Michael Landgrebe

**Affiliations:** 1 Institute of Neuroscience and Medicine–Neuromodulation, Research Center Jülich, Leo-Brand-Straße, 52425, Jülich, Germany; 2 Department of Neuromodulation, University of Cologne, Kerpener Straße, 62, 50924, Cologne, Germany; 3 Department of Psychiatry and Psychotherapy, University of Regensburg, Universitätsstraße 84, 93053, Regensburg, Germany; 4 Interdisciplinary Tinnitus Clinic, University of Regensburg, Universitätsstraße 84, 93053, Regensburg, Germany; 5 Center for Clinical Studies, University Hospital Regensburg, Franz-Josef-Strauss-Allee 11, 93053, Regensburg, Germany; 6 Department of Psychiatry and Psychotherapy, Sozialstiftung Bamberg, Buger Straße 80, 96049, Bamberg, Germany

**Keywords:** Tinnitus, Tinnitus questionnaire, Minimal clinically important difference, Clinical significance, Receiver operating characteristic

## Abstract

**Background:**

Development of new tinnitus treatments requires prospective placebo-controlled randomized trials to prove their efficacy. The Tinnitus Questionnaire (TQ) is a validated and commonly used instrument for assessment of tinnitus severity and has been used in many clinical studies. Defining the Minimal Clinically Important Difference (MCID) for TQ changes is an important step to a better interpretation of the clinical relevance of changes observed in clinical trials. In this study we aimed to estimate the minimum change of the TQ score that could be considered clinically relevant.

**Methods:**

757 patients with chronic tinnitus were pooled from the TRI database and the RESET study. An anchor-based approach using the Clinical Global Impression (CGI) scale and distributional approaches were used to estimate MCID. Receiver Operating Characteristic (ROC) curves were calculated to define optimal TQ change cutoffs discriminating between minimally changed and unchanged subjects.

**Results:**

The relationship between TQ change scores and CGI ratings of change was good (r = 0.52, p < 0.05). Mean change scores associated with minimally better and minimally worse CGI categories were −6.65 and +2.72 respectively. According to the ROC method MCID for improvement was −5 points and for deterioration +1 points.

**Conclusion:**

Distribution and anchor-based methods yielded comparable results in identifying MCIDs. ΔTQ scores of −5 and +1 points were identified as the minimal clinically relevant change for improvement and worsening respectively. The asymmetry of the MCIDs for improvement and worsening may be related to expectation effects.

## Background

Subjective tinnitus is a frequent sensation of sound that cannot be attributed to an external sound source [1,2]. Treatment of tinnitus is difficult and for most of the currently used treatment strategies the evidence of efficacy is low [3]. Many interventions in reducing tinnitus-related distress are based on cognitive theories of behavior regulation and on psychological treatments [4,5]. In recent years animal models and neuroimaging of tinnitus perception have contributed to substantial advances in the understanding of the pathophysiology of tinnitus [2,6-8], which in turn has prompted the development of new treatment strategies [4,9-11]. For assessing the efficacy of the various tinnitus treatment strategies, prospective placebo-controlled randomized trials have to be performed. An important aspect in the design of such clinical trials is the choice of the outcome measure. However in tinnitus research, the quantification of tinnitus severity can be challenging for several reasons. First, tinnitus is a purely subjective phenomenon and lacks any objectively identifiable variables or markers. Second, taking into account that tinnitus affects many different aspects of well-being (i.e., sleep, mood, concentration, energy), different patients may be more bothered by some symptoms and less by others [12].

In the evaluation of new treatments for tinnitus, several instruments are used to provide a quantification of tinnitus symptoms [13]. The TQ is a widely used questionnaire for the quantification of tinnitus complaints. Developed by Hallam et al. [14], it has been translated and validated in German language [15] and is widely used in the German version. The TQ incorporates scales evaluating emotional and cognitive distress, intrusiveness, auditory perceptual difficulties, sleep disturbances, and associated somatic complaints. It was recommended, among other validated questionnaires, such as Tinnitus Handicap Inventory (THI) [4], Tinnitus Handicap Questionnaire (THQ) [16], and Tinnitus Reaction Questionnaire (TRQ) [17], in a consensus document of the Tinnitus Research Initiative (TRI) to be used as an outcome measurement in clinical trials [13]. It has also been used, in isolation or in conjunction with other tinnitus questionnaires, for assessing the effect of various therapeutic interventions in many clinical studies on chronic tinnitus [9,18-20].

When using health status questionnaires to ascertain whether a treatment for a given condition is effective or not, statistical significance of effects is usually reported. Statistically significant effects are those that are beyond a certain level of chance. However, noteworthy statistical significance of a treatment effect largely depends on the sample size and does not provide information of whether observed changes are clinically meaningful. In contrast, clinical relevance of a treatment effect refers to its impact upon the patient, to its implications for management of the patient, to its ability to meet standards of efficacy set by patients, clinicians, and researchers [21,22]. The question of what is the clinical meaning of the reported score change usually remains open [21-23]. Thus, deriving clinical meaning from statistically significant results may be misleading. Results of clinical studies for tinnitus usually report changes in TQ scores as continuous variables for each group [24] (e.g., mean change or Effect Size (ES) for each group) and thus are difficult to interpret when translated to the level of clinical relevance and an individual response. Therefore, exact knowledge about which change of the TQ is clinically relevant is critical, both for the design and interpretation of a clinical trial as well as for rational decision-making in clinical management of tinnitus patients. In order to estimate meaningfulness of changes in clinical scores, the concept of the Minimal Clinically Important Difference has been developed [25,26]. The MCID for a given questionnaire score can be defined as the value above which the change becomes clinically relevant.

However, no consensus exists on the methods that should be used in estimating the MCID [25]. Techniques used in MCID evaluation are usually divided in two groups: distribution- and anchor-based. Distribution-based methods use statistical properties of a study's results, e.g., ES, Standard Error of Measurement (SEM) and other measures obtained from characteristics of study population [21-23,27,28]. In anchor-based methods changes in used measuring instrument (e.g., patient reported outcomes, PRO) are referenced to an anchor [21,25,26,29], which should reflect the patient’s perspective [30]. This is especially relevant for a purely subjective condition such as tinnitus [31].

Given that tinnitus is a purely subjective condition a patient-rated CGI seems more appropriate as a judgment for the change of tinnitus-related global impairment than a CGI of a rater, based on an interview. Accordingly, several recent clinical trials used a patient-rated CGI change as outcome criterium [32,33]. Therefore, patient-rated CGI scales represent a valid example of a reference anchor [26,34,35]. Anchor based methods were recommended as primary methods for MCID estimation complemented by various distribution-based estimates (e.g., ES and SEM) as supportive information [25,36,37].

It has been implied in the available literature that the magnitude of a meaningful score change may be independent of the direction of change, i.e., MCID for improvement (MCID-I) is equivalent to the MCID for deterioration (MCID-D) [26,38]. However, clinical experience and previous studies challenge this assumption [37,39]. Thus MCID can be bidirectional and can differently reflect an improvement and deterioration. It has been observed in studies in tinnitus and chronic pain patients that a smaller change in measuring instrument scores is sufficient to feel deterioration than the change needed to feel improvement [31,39,40]. In contrast studies in cancer patients showed the opposite. Small improvements were considered relevant by patients whereas declines have to be large to be perceived as worsening [37,41]. 

The aim of this study was to determine which change in the TQ score is considered as a meaningful clinical change with the main aim to identify the minimal score reduction which is perceived as improvement (MCID-I) [37,39]. We defined MCID as the smallest change in the measurement instrument used that signifies a perceptible improvement or deterioration in tinnitus from the patient’s perspective. To estimate clinically relevant changes, we analyzed data from the TRI database [42] and from the RESET study [43]. We compared changes in the TQ with patient’s subjective evaluation of treatment-related changes of tinnitus assessed with the Clinical Global Impression Change scale.

## Methods

### Patients

Data from the RESET study (ClinicalTrials.gov Identifier: NCT00927121) [43] combined with data from the TRI database [42] were analyzed. The TRI database contains longitudinal data collected in a standardized way from tinnitus patients undergoing different types of treatment interventions in different study centers and different countries. Collection of data for the TRI database was approved by the local ethics committee of the University of Regensburg, Germany. The RESET study was a multicentric randomized, controlled clinical trial on acoustic CR neuromodulation in the treatment of chronic tinnitus, performed in Germany between 2009 and 2010, ethical committee approved the trial design and all changes.

757 patients (694 from the TRI database and 63 from the RESET study) from 7 different centers in Germany who had received different forms of tinnitus treatment, including acoustic coordinated reset neuromodulation, transcranial magnetic stimulation, behavioral therapy and pharmacologic treatment were included in the analysis. Data for every patient included the TQ at baseline, TQ and CGI at outpatient visits and at the end of treatment. The number of visits between the begin and the end of treatment was 1 for 112 patients, 2 for 74 patients, 3 for 151 patients, 4 for 29 patients, 5 for 134 patients and 6 for 257 patients (total 3041 visits). The mean time between baseline and assessment was 44 days.

### Questionnaires and scales

Tinnitus severity was assessed with the German version of the Tinnitus Questionnaire [15]. The German version of the TQ consists of 52 questions and the total sum score ranges from 0 (no distress) to 84 (very severe distress). The total score is based on 42 questions out of 52 and two question is included twice. This questionnaire indicates the general level of tinnitus related psychological and psychosomatic distress. Factor analysis of the German version of the TQ revealed the factors emotional and cognitive distress, intrusiveness, auditory perceptual difficulties, sleep disturbances, and associated somatic complaints*.* According to its total score, the TQ is divided in four distress levels: mild (0–30), moderate (31–46), severe (47–59), very severe (60–84) [15].

Patient-rated global assessment of treatment effects was performed in all patients of this study. In the RESET study, a custom designed Clinical Global Impression (CGI) score was used. In this CGI at each visit, patients were asked to give a verbal categorical rating of their tinnitus loudness and annoyance for each ear where tinnitus was perceived as compared to baseline. Patients had 5 choices: 1 = much better; 2 = somewhat better; 3 = no change; 4 = somewhat worse, or 5 = much worse. CGI was side specific. Patients were not permitted to refer to any previous markings. Reduction of subjectively perceived tinnitus loudness and reduction of tinnitus annoyance were shown to be highly correlated [43,44]. Furthermore, a robust correlation between the reduction of tinnitus loudness and reduction of TQ scores was reported [43]. Therefore, at each visit the mean score of all CGI values (from both sides, in case of bilateral tinnitus, and from both domains: loudness and annoyance) served as the summary global rating of change for each patient.

A CGI-I was applied in the studies included in the TRI database, to assess a patient’s subjective perception about the change of tinnitus over time [31]. Patients had to mark 1 of the 7 answers: 1 = very much better; 2 = much better; 3 = minimally better; 4 = no change; 5 = minimally worse; 6 = much worse, and 7 = very much worse. In both, the TRI database and the RESET study, the patients were asked to “rate the improvement of their tinnitus complaints compared to before the beginning of treatment”.

For better applicability across TRI database and the RESET study we combined “very much better” and “much better” categories of the CGI-I used in the TRI database into a “much better” as well as “much worse” and “very much worse” in to a “much worse” categories. In addition CGI-I categorical numeration of the CGI-I used in the TRI database was changed to be in accordance with the 5 level CGI similar to the one used in the RESET study.

For analysis, different groups were formed according to the CGI scores:

Much better: CGI = 1

Minimally better: CGI = 2

No change: CGI = 3

Minimally worse: CGI = 4

Much worse: CGI = 5

### Statistical analysis

TQ change from baseline was determined by subtracting the value at visit from baseline value. Thus, negative TQ change value meant a reduction of tinnitus. A one-way analysis of variance (ANOVA) of the TQ changes was conducted to test for significant differences between CGI levels. To compare 2 continuous variables, the t-test for independent or dependent variables was used. All tests were two-tailed and exploratory, i.e., no adjustments for multiple comparisons were performed and a p < 0.05 was considered statistically significant. Missing data were not replaced. MCID calculations should be based on the patient-reported outcomes, e.g., TQ, that are correlated at r ≥ 0.30 with appropriate patient based or clinical anchors, e.g., CGI [45]. Thus we assessed the usefulness of the used anchor (i.e., CGI) by calculating the Spearman rank correlation of CGI scores with the absolute and relative ((i.e., visit value-base line value)/base line value) changes of the TQ scores for the comparison of the two. Intraclass correlation (ICC) was used to determine test–retest reliability of the CGI in patients whose TQ score change between two visits was 2 points or less in any direction. First and second visit data from the TRI database and 8 and 12 weeks visit data from the RESET study were used to calculate ICC. To estimate the ES for TQ score changes at different levels of CGI Cohen’s d was used. It was calculated using the original standard deviations formula: d = (M_1_ – M_2_)/SD_pooled_ where M_1_ and M_2_ are the mean values of TQ at baseline and visits, and SD _pooled_ is the pooled standard deviation for independent samples [27]. The Standard Error of Measurement for the TQ was also computed: SEM = SD$\sqrt{1-r}$, where SD is the standard deviation of TQ scores, and r is the test-retest reliability of the TQ, i.e., 0.94.

ROC curves were calculated to define cutoff values for TQ changes that best distinguished those who had minimally improved or minimally deteriorated from those who had not changed [46,47]. A ROC TQ plot was produced by plotting true positive rate, or sensitivity vs. 1-specificity or false positive rate. Sensitivity was defined as the number of patients correctly identified by selected measure or test, e.g., TQ threshold, as changed divided by number of all patients who truly underwent a change. Specificity was defined as the number of patients who were correctly classified by the selected measure as not changed divided by the number of all patients who truly did not undergo an important change. The optimal amount of TQ change that was used to discriminate between subjects rated as minimally improved or minimally worse from subjects rated as unchanged on the CGI was selected as corresponding to the highest average of sensitivity and specificity. Statistical analyses were performed in STATISTICA 8 software (http://www.statsoft.com). The ROC statistic was calculated using the method implemented in the commercially available software MedCalc (http://www.medcalc.org). Positive likelihood ratio was defined as ratio between sensitivity and 1-specificity. Negative likelihood ratio was defined as ratio between 1-sensitivity and specificity.

To investigate the time course of mean TQ change in the minimally better CGI level with time passed from baseline we divided all visits in to 11 categories: 1–9, 10–19, 20–29, 30–39, 40–49, 50–59, 60–69, 70–79, 80–89, 90–99 and ≥ 100 days since baseline. For each of the 11 categories a mean TQ change value in patients who indicated “minimally better” on the CGI was calculated. In case that one patient had multiple observations in one of the 11 periods, the mean value was calculated.

## Results

**Figure 1:**
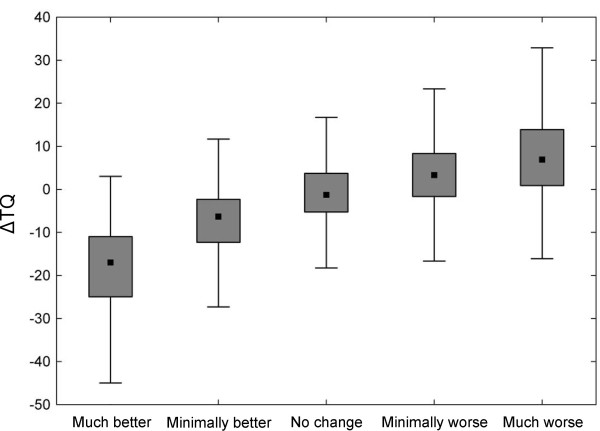
**Boxplots of Tinnitus Questionnaire score changes from baseline categorized by the Clinical Global Impression.** Median value, 1^st^ and 3^d^ quartile and the non-outlier range are shown.

757 patients from Germany, aged between 12 and 86 years, were included in this study; the mean tinnitus duration was 7.5 years (min 2 months, max 44 years). 751 patients suffered from chronic subjective tinnitus (> 3 month) and 6 had tinnitus duration between 2 and 3 months. Baseline characteristics are presented in Table 1. The average age of the patients was 50.8 (SD 12.4), and 71.1% of them were men. The mean baseline score of the TQ was 42.0 (SD 16.9). The distribution of the changes of the TQ scores, categorized by the CGI is given in Figure 1. Number of patients in each treatment category were as follows: transcranial direct current stimulation 28, transcranial magnetic stimulation 562, pharmacological treatment 35, CR neuromodulation 46, noisy CR neuromodulation 12, placebo 37, transcutaneous vagus nerve stimulation 24, and for 13 patients there was no record. GCI test-retest reliability was found to be very good: ICCs for the seven item CGI used in TRI database and five item CGI used in the RESET study were 0.74 (95% CI 0.63/0.82) and 0.72 (95% CI 0.36/0.87) respectively. We did not observe any consistent trends indicating a progressive change of MCID with time passed since baseline (Table 2). Based on this observation all further analyses were performed on data from the last outpatient visit for every patient.

**Table 1:** Patient baseline characteristics (N = 757)


Age, years (SD)		50.8 (SD 12.4)
Tinnitus duration, years (SD)		7.5 (SD 8.0)
Gender, N (%)		
	Male	538 (71.1%)
	Female	219 (28.9%)
Laterality of tinnitus N (%)		
	Right	92 (12.1%)
	Left	145 (19.2%)
	Bilateral/inner head	520 (68.7%)
TQ at baseline, Mean (SD)		42.0 (SD 16.9)
Tinnitus severity at baseline based on TQ, N (%)		
	Severe	136 (18.0%)
	Moderate	170 (22.4%)
	Mild	234 (30.9%)
	Slight	217 (28.7%)

Abbreviations: SD, standard deviation.

**Table 2:** Mean TQ change in the minimally better CGI level with time passed from baseline (no significant differences neither clear trend)

**Date range (days)**	**Number**	**Mean (SD)**
1-9	119	−3.28 (6.26)
10-19	171	−4.87 (6.53)
20-29	130	−4.82 (7.74)
30-39	9	−9.67 (12.44)
40-49	1	−11.00 (N/A)
50-59	91	−5.32 (8.99)
60-69	14	−4.43 (9.75)
70-79	10	−6.00 (12.46)
80-89	103	−5.77 (7.23)
90-99	38	−7.68 (6.66)
≥ 100	10	−4.47 (7.19)

One way ANOVA revealed significant differences between the TQ mean score changes across 5 CGI levels (F = 79.5, p < 0.001). The distribution of TQ changes and ES corresponding to each CGI category are summarized in Table 3. The Spearman correlation coefficient between absolute TQ change and CGI was r = 0.52 (p < 0.001) and between relative TQ change and CGI was r = 0.42 (p < 0.001). The minimally better group had a mean TQ change of −6.65 (95% CI −7.90/-5.39) and an Effect Size of 0.41 (95% CI 0.18/0.63). The minimally worse group had a mean TQ change of 2.72 (95% CI 0.95/4.49) and an ES of −0.14 (95% CI −0.39/0.11). CGI no change group had a mean change −0.33 (95% CI −1.19/0.54) that was not significant as compared to baseline (p = 0.46).

**Table 3:** TQ characteristics of clinical global impression groups

**CGI**	**Number (%)**	**ΔTQ, Mean (SD)**	**95% CI of ΔTQ**	**Cohen d**	**95% CI of d**
Much better	79 (10.4)	−16.73 (12.65)	−19.57/-13.90	0.96	0.63/1.29
**Minimally better**	**158 (20.9)**	**−6.65 (7.99)**	**−7.90/-5.39**	**0.41**	**0.18/0.63**
No change	357 (47.2)	−0.33 (8.30)	−1.19/0.54	0.02	−0.13/0.17
**Minimally worse**	**119 (15.7)**	**2.72 (9.76)**	**0.95/4.49**	**−0.14**	**−0.39/0.11**
Much worse	44 (5.8)	6.20 (11.35)	2.75/9.66	−0.36	−0.78/0.06

It has been previously reported that MICD values are dependent on baseline values (e.g., in patients with low back pain) [48]*.* In Table 4 mean changes from the 5 CGI levels were classified according to the TQ baseline score. Patients with higher TQ scores at baseline (indicating more severe complaints), generally needed greater reduction of the TQ to be classified as minimally importantly improved based on the used anchor. For worsening of tinnitus symptoms this seemed not to hold true and even a slight opposite tendency was observed, i.e., patients with higher TQ scores at baseline, generally needed lower increase of the TQ score to feel minimally worse.

**Table 4:** Dependence of TQ change in CGI 2 on baseline value

**CGI**	**0-30**		**31-46**		**47-59**		**60-84**	
	**N**	**Mean (SD)**	**N**	**Mean (SD)**	**N**	**Mean (SD)**	**N**	**Mean (SD)**
Much better	30	−8.47 (8.05)	18	−16.39 (10.09)	19	−24.95 (11.14)	12	−24.92 (15.05)
Minimally better	54	−3.17 (7.15)	46	−7.07 (6.86)	42	−8.86 (9.02)	16	−11.38 (6.44)
No change	111	0.98 (7.68)	118	0.29 (9.03)	64	−0.56 (8.04)	64	−3.50 (7.51)
Minimally worse	35	3.20 (9.10)	39	3.49 (9.51)	22	2.23 (14.20)	23	1.17 (5.51)
Much worse	7	12.86 (13.52)	13	7.23 (10.00)	15	5.00 (12.15)	9	1.56 (8.95)

**Figure 2:**
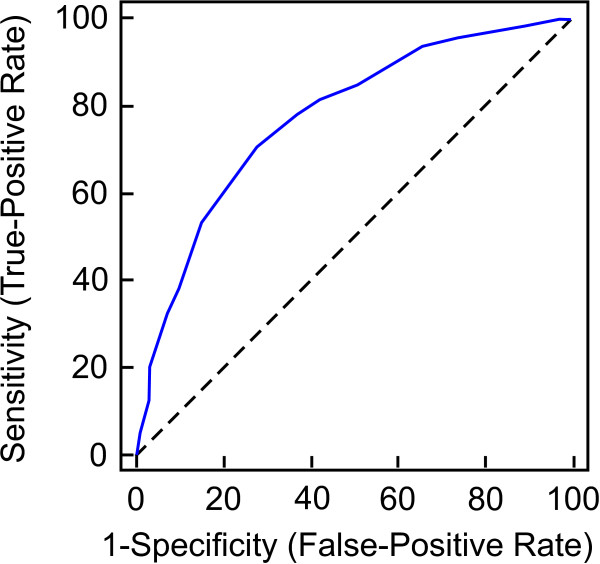
**Receiver Operating Characteristic curve.** Plot represents comparison of two operating characteristics, i.e., False-Positive Rate and True-Positive Rate as the criterion of TQ change.

**Figure 3:**
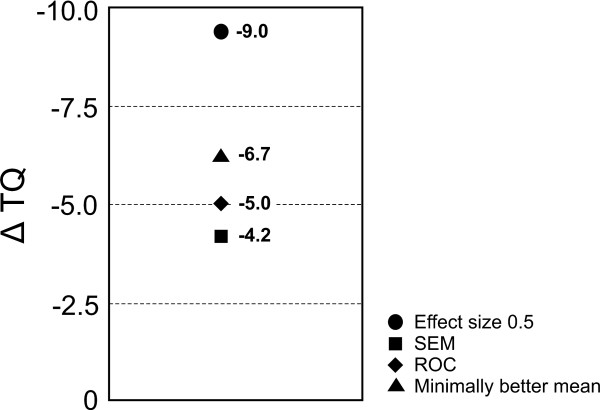
Summary of distribution- and anchor-based estimates of MCID.

Table 5 provides specific threshold levels (optimal cut-off points), generated from the ROC analyses for all patients from the CGI minimally better change group and for these patients grouped according to their baseline TQ scores. The area under the ROC curve for all TQ scores was 0.79 (95% CI 0.78-0.81) for minimally better vs. no change groups (Figure 2) and 0.60 (95% CI 0.55-0.64) for minimally worse vs. no change groups. The optimal amount of TQ change that was used to discriminate between the minimally better group and the no change group was −5 points and +1 point between the minimally worse group and the no change group. However, a relatively low area under the ROC curve (0.60) as well as low sensitivity (56.30) and specificity (61.34) values for the minimally worse group vs. the no change group make these estimates less reliable then the ones obtained for the minimally better group vs. the no change group. These results also indicate that higher threshold values were obtained for the patients with higher TQ scores at baseline for the minimally better vs. the no change group. The SEM was 4.2 points and was slightly smaller than ROC based MCID-I estimate and mean TQ change for symptoms reduction. An effect size of d = 0.5 was proposed as a “universal” cut off point in the interpretation of changes of quality-of-life data [49]. The mean pooled standard deviation of all data is 18. With an estimated standard deviation of the before-after difference of the TQ score = 18 the effect size of d = 0.5 can be calculated to be ΔTQ = 9. Figure 3 provides a summary of MCID-I estimates defined using different methods.

**Table 5:** Test characteristics for best Tinnitus Questionnaire cutoff point for the minimally better group

**TQ**	**N minimally better group**	**ROC MCID Estimate**	**Area under ROC Curve (95% CI)**	**Sensitivity ** **(95% CI)**	**Specificity** **(95% CI)**	**Positive likelihood ratio (95% CI)**	**Negative likelihood ratio (95% CI)**
**0-84**	**158**	**−5**	**0.79 (0.78-0.81)**	**71.43 (63.4/78.6)**	**73.10 (67.9/77.9)**	**2.66 (2.4/3.0)**	**0.39 (0.3/0.5)**
0-30	54	−2	0.65 (0.61-0.68)	66.67 (52.5/78.9)	59.46 (49.7/68.7)	1.64 (1.3/2.1)	0.56 (0.4/0.9)
31-46	46	−3	0.74 (0.70-0.78)	80.43 (66.1/90.6)	60.17 (50.7/69.1)	2.02 (1.6/2.5)	0.33 (0.2/0.6)
47-59	42	−5	0.75 (0.71-0.79)	71.43 (55.4/84.3)	64.06 (51.1/75.7)	1.99 (1.5/2.6)	0.45 (0.2/0.8)
60-84	16	−12	0.80 (0.76-0.84)	62.50 (35.4/84.8)	89.06 (78.8/95.5)	5.71 (3.9/8.4)	0.42 (0.2/1.1)

As the analysis was based on 2 datasets in which 2 different CGI questionnaires were used, we were interested in how far this could have affected the results. Medians of the CGI minimally better group were −7 and −6 for the RESET and TRI datasets. Furthermore, CGI minimally better groups from the RESET and TRI samples were not significantly different (p = 0.18). Thus combining data from 2 datasets (i.e., TRI and RESET) is not likely to have affected the final result.

## Discussion

To improve the quality of tinnitus management and to evaluate new tinnitus therapies validated tinnitus specific questionnaires are used. The assessment of clinical significance of changes in these questionnaires often poses the biggest challenge in interpretation of obtained results. Because of its validity and reliability the TQ is a powerful tool in the field of tinnitus research and management [13,15]. Moreover, its widespread use, especially in German speaking countries, enables comparisons across clinical trials. However no empirical data are available to judge which minimal change is needed to be of clinical relevance. In this study we addressed this issue by evaluating a MCID-I and MCID-D for the TQ.

The data analyzed in this study come from a large cohort of patients that underwent various treatment interventions in different centers, ranging from primary to tertiary referral centers. All patients were assessed with similar measurement instruments and methodology. The large dataset was collected in multiple centers including a wide range of patients with different treatments; therefore, it can be regarded as representative and the obtained results can be generalized to other samples.

Techniques available for evaluating MCID are usually divided in two groups: distribution- or anchor-based [21]. It has been recommended to estimate the MCID based on several methods using relevant anchors complemented by various distribution-based estimates (i.e., ES, SEM) as supportive information, and then triangulate on a single value or small range of values for the MCID based on all used methods [36,37]. Accordingly, we used the CGI as the self-assessment of the changes in tinnitus symptoms to investigate the size of a meaningful change in TQ score. Significant association between patient’s global retrospective rating and the actual change of TQ scores as shown by correlation between these two instruments enabled a comparison of the two.

The smallest degree of meaningful change is represented by minimally worse and minimally better CGI levels. Some studies de-emphasize an important distinction between improvement and worsening of patient’s symptoms [26,38]. In observations rated as minimally worse and minimally better mean changes in TQ scores were consistent with the expected direction of change: ΔTQ +2.72 and −6.65 respectively. These findings are also consistent with previously published data on the other validated tinnitus questionnaire, i.e., Tinnitus Handicap Inventory [31]. Among patients who reported a minimal worsening of their tinnitus symptoms, average change scores on the TQ were smaller then among patients who reported minimal improvement, indicating that it took a smaller amount of TQ score increase for patients to perceive their tinnitus as worse.

The results for the much better and much worse groups, i.e., ΔTQ −16.7 vs. +6.20 respectively, are consistent with the changes in the minimally improved and worsened groups in the sense that higher score changes are needed for meaningful improvement than for subjectively perceived worsening. Consistent with this asymmetry is the finding that the group, that observed “no change”, had an average improvement of −0.33 points in the TQ. This response bias might reflect the anticipation of improvement. With an expectation of improvement, a small increase of symptoms is sufficient to be perceived as worsening whereas the symptom reduction has to be more pronounced, to be really perceived as relevant. Another general methodological concern of MCID estimation is reliability of clinical global change ratings over time as patients internal reference may change with time or be influenced by recall bias [50]. In our study we investigated, if the mean ΔTQ for minimally improved patients changes in some consistent manner with time passed since baseline. Our results showed that the mean TQ change in the minimally better group during the first 10 days after the baseline measurement did not differ from those made after 100 days passed since baseline. Even though this study was not designed to investigate changes of patient’s internal frame of reference with time, our results do not show evidence of any consistent changes of such an internal reference. Accordingly we do not expect that longer or shorter intervals between baseline and MCID assessment, as compared to mean interval in this study, i.e., 44 days, could have resulted in a significantly different MCID estimate. In this study, four different MCID-I estimates were computed (Figure 3). The strategy used in the ROC analysis for MCID estimation was to maximize both sensitivity and specificity. The results show that the MCID-I magnitude for all patients combined was −5 TQ points. However, the magnitude of a TQ change that was perceived by the patients as minimal reduction or worsening of tinnitus depended on patients' baseline TQ scores (Tables 4 and 5). For example, patients with high initial TQ scores needed to undergo an improvement of approximately −12 TQ points in order for the change to be judged important. Whereas for patients with low TQ scores an improvement of −2 TQ points suffices in order to be perceived as improvement.

There are 2 possible explanations for this trend. First, it may be that more disabled patients were only satisfied with greater reduction of tinnitus. It has been suggested previously that the thresholds for change may shift depending on initial symptoms severity and that smaller improvements may mean more to the patients with mild rather than severe disability [51,52]. Second, some patients have achieved greatest possible reduction on TQ score and no further effect on tinnitus was possible – a so-called ”ceiling effect”. From the entire population only one patient who reported minimally better achieved minimal (zero) TQ score. This patient had a TQ change of −3 points. No patient achieved maximal (eighty four) TQ score. Both of these reasons probably contribute to the finding that the MCID depends on the baseline tinnitus severity score. Unfortunately the magnitude of contribution of each of these factors cannot be determined. MCID-I estimate based on the mean TQ change of the minimally better group was around −6.65 points and was very similar to the one defined by the ROC method. These results are consistent with previously published MCID estimates based on clinical experience and a distribution-based approach using a small data set [53]. SEM has been proposed as a useful method of expressing the imprecision of an instrument and its responsiveness and was used in several studies [28,54]. In our study SEM was 4.2 and was smaller than ROC and mean change estimates of MCID-I. However SEM is related to the minimal statistical detectable difference and not to the individually perceived benefit from the treatment. The ES of the TQ changes for the minimally better group was 0.41. Thus using ES d = 0.5 as proposed by Norman et al. would represent the most conservative MCID-I estimate of all (Figure 3) [49]. Similarly to SEM, ES also do not represent a clinically meaningful estimate of MCID change as it provides no direct information about the MCID and is a way of expressing the observed change in a standardized way. The group, which reported highly relevant improvement, had a mean reduction in TQ of about −16.7 points and may be considered a “super response” group. This closely corresponds to the ”super response“ of 15 points in TQ proposed by Goebel et al. [55] that was used in clinical studies [43].

Based on our results the MCID-I estimated as a ΔTQ of −5 points seems an acceptable choice. This also closely corresponds to the cut-off values proposed in the past to interpret results of clinical studies and to define a therapeutic success (response) [43,53,55]. A change of ≈ −17 points would represent a “super response”, however more detailed analysis is needed to define the cut off point for a ,,super response“. Calculation of the proportion of patients who achieve this magnitude of relief, i.e., -5 TQ points, would provide clinically relevant information about the efficacy of the evaluated treatment [56]. By using MICD as a response criterion one can also estimate the proportion of responders in each trial arm and thus calculate the number of patients needed to treat for further trials. This approach may be used complementary to defining statistical significance of group differences where in large samples even small changes in mean scores can yield “statistically significant” results that may be without clinical relevance.

## Conclusions

MCID of the change in TQ score was found to be around −5 points for improvement and +1 point for deterioration. These results provide an orientation for what a clinically meaningful change in the TQ score is. MCID may serve as an orientation to interpret changes in individual patients as well as in results of clinical trials. We investigated the influence of baseline scores on MCID. The magnitude of an important change depended on patient’s baseline TQ scores. However, the role of other potential influencing factors, e.g., duration or etiology of tinnitus should also be investigated in further studies.

## Competing interests

C. Hauptmann and P. A. Tass have a contractual relationship with ANM Adaptive Neuromodulation GmbH. I. Adamchic, B. Langguth, M. Koller, M. Schecklmann, F. Zeman, M. Landgrebe declare that they have no competing interests.

## **Authors contribution**

**IA** conceived the idea of the study, designed the study, acquired data (RESET study), performed statistical analysis, interpreted data and drafted the manuscript. **PAT** headed the RESET study, acquired data (RESET study), interpreted data, drafted the manuscript, final approval. **BL** acquired and interpreted data and drafted the manuscript, final approval. **CH** acquired data (RESET study), interpretation of results, reviewing paper, final approval. **MK** conceptualizing the TRI database, acquired data (TRI database), reviewing paper, final approval. **MS** acquired data (TRI database), reviewing paper, final approval. **FZ** maintaining the TRI database, interpretation of statistical analysis, drafted the manuscript, final approval. **ML** interpretation and acquisition of the data (TRI database), reviewing paper, final approval. All authors read and approved the final manuscript.
